# Genome-Wide Association Study Reveals Genetic Loci, Candidate Genes and Favorable Haplotypes for Important Agronomic Traits in *Auricularia cornea*

**DOI:** 10.3390/jof12030186

**Published:** 2026-03-05

**Authors:** Xu Sun, Lixin Lu, Fangjie Yao, Ming Fang, Xiaoxu Ma, Yuling Cui, Jian Sun, Xianqi Shan, Wei Liu

**Affiliations:** 1 Internationally Cooperative Research Center of China for New Germplasm Breeding of Edible Mushroom, Jilin Agricultural University, Changchun 130118, China; sunxu0512@163.com (X.S.); lixinl@jlau.edu.cn (L.L.); 13595635906@163.com (Y.C.); sj121316sj@163.com (J.S.); 2 Lab of Genetic Breeding of Edible Mushroom, College of Horticulture, Jilin Agricultural University, Changchun 130118, China; fangming@jlau.edu.cn (M.F.); maxiaoxu54@163.com (X.M.); shanxianqi2022@163.com (X.S.); 13314354969@163.com (W.L.)

**Keywords:** *Auricularia cornea*, genome-wide association study (GWAS), haplotype

## Abstract

*Auricularia cornea* is rich in nutrients and bioactive compounds, and with the continuous expansion of its industrial cultivation, elucidating the genetic basis of key agronomic traits is essential for marker-assisted breeding and cultivar improvement. In this study, 140 *A. cornea* germplasm accessions representing diverse geographic origins were subjected to a comprehensive two-year phenotypic evaluation of ten agronomic traits, including FBL, FBW, FBT, Yield, MGR, GP, and fruiting body color. The results showed that all traits exhibited substantial genetic variation across the population, and most traits displayed high heritability. Genome-wide association studies (GWAS) identified 1178 and 821 SNPs significantly associated with agronomic traits in the two respective years, with yield-related loci predominantly located on chromosome 9. Based on an integrated significance threshold, ten core SNPs were ultimately retained, and 42 putative candidate genes were identified within ±5kb flanking regions of these SNPs. These candidate genes were mainly involved in cell wall polysaccharide modification, redox regulation, pigment biosynthesis, metabolic processes, and signal transduction. Furthermore, haplotype analysis identified six superior haplotypes associated with ear morphology, yield, MGR, and GP, and accessions carrying these superior haplotypes exhibited significantly enhanced phenotypic performance. Overall, this study provides a systematic dissection of the genetic architecture of important agronomic traits in *A. cornea* and offers a solid theoretical foundation for high-yield and high-quality molecular breeding and genetic improvement.

## 1. Introduction

*Auricularia cornea* is one of the most important edible and medicinal fungi in China, possessing both notable nutritional value and functional bioactivities, and its fruiting bodies are rich in polysaccharides [1] with reported antithrombotic [2], anticoagulant [3], antitumor [4], and anti-aging [5] effects. Agronomic traits such as yield, morphological characteristics, growth duration, and coloration directly determine the commercial quality and cultivation efficiency of *A. cornea* [6]. With the continuous advancement of large-scale and standardized production, there is an increasing demand for high-yield, high-quality, and stable cultivars. However, most key agronomic traits of *A. cornea* are typical quantitative traits controlled by multiple genes and strongly influenced by environmental factors, and their improvement has long relied on empirical phenotypic selection, thereby limiting breeding efficiency and the precision of genetic improvement [7].

In recent years, genome-wide association studies (GWAS) have become an important approach for dissecting the genetic basis of complex quantitative traits [8,9] and have successfully identified genetic loci and candidate genes associated with yield, morphology, and quality in a wide range of crops, including maize [10,11], wheat [12,13], rice [14,15], and soybean [16,17]. In edible fungi, genetic loci related to fruiting body morphology, yield traits, and stress resistance have been successfully identified in *Lentinula edodes* [18]. Compared with crop studies, research on gelatinous edible fungi such as *A. cornea* remains markedly insufficient in terms of systematic phenotypic characterization, multi-trait joint association analysis, and functional mining of candidate genes, particularly with respect to genome-wide genetic dissection of multiple key agronomic traits. Moreover, most existing studies are confined to the statistical association of significant loci, lacking in-depth analyses of haplotype structures in key genomic regions and their relationships with phenotypic variation, which limits the practical application of association results in molecular breeding.

Based on this background, this study used a natural population of 140 *A. cornea* strains as research materials, systematically measured multiple important agronomic traits, conducted genome-wide association analyses to identify genetic loci significantly associated with key traits, and further combined haplotype analysis to elucidate the genetic structural characteristics of candidate regions, thereby screening potential candidate genes. This study aims to clarify the genetic regulatory basis of important agronomic traits in *A. cornea*, provide a theoretical foundation for functional gene mining and molecular marker development, and ultimately lay a genetic basis for precision breeding and the selection of superior cultivars.

## 2. Materials and Methods

### 2.1. A. cornea Strains Used for Sequencing and Cultivation Experiments

A total of 140 *A. cornea* strains representing diverse geographic origins were selected for genome sequencing and cultivation-based phenotypic evaluation in this study. Detailed information on strain identification numbers, types of strains, and origins is provided in Table S1.

### 2.2. Cultivation Experiments

Two independent cultivation experiments were conducted in 2024 and 2025 at Jilin Agricultural University. All strains were cultivated in mushroom houses under a randomized block design, and greenhouse environmental conditions were uniformly regulated using the Nongxin agricultural environmental monitoring and control platform, with the average temperature maintained at 27 °C and the average relative humidity at 85% during the experimental period. Here, 10 fruiting body-related traits (FBRTs) were investigated, including mycelial growth rate (mm d^−1^) (MGR); growth period, defined as the time from inoculation to the first harvest (d) (GP); predominant ventral color of fresh ear pieces (VCF); dorsal color of fresh ear pieces (DCF); ventral color of dried ear pieces (VCD); dorsal color of dried ear pieces (DCD); fruiting body length (mm) (FBL); fruiting body width (mm) (FBW); fruiting body thickness (mm) (FBT); and yield. All traits were measured using 30 replicates for each strain [19] (Table S2).

### 2.3. Phenotypic Data Analysis

Commercial traits of *A. cornea* were assigned scores and processed according to specific criteria, as detailed in Table S3. Quantitative traits were directly analyzed using the original measured data. Microsoft Excel was used to organize the raw data and to calculate the class frequency of each trait, as well as the Shannon–Wiener diversity index (H′) and Simpson’s genetic diversity index (D), where H′ = −∑[(pᵢ) × ln(pᵢ)] and D = 1 − ∑pᵢ^2^, with pᵢ representing the proportion of trait i relative to the total [20]. The coefficient of variation (CV, %) was used to describe the degree of dispersion of phenotypic traits and was calculated as CV = S/x, where S is the standard deviation and x is the mean. Basic statistical parameters, including mean, maximum, minimum, standard deviation, variance, and coefficient of variation, were calculated using SPSS 25.0 software [21]. Pearson correlation analysis and R-type cluster analysis were also performed using SPSS, and all analyses were conducted on z-score-standardized data based on the mean value of each trait for each accession [22].

### 2.4. SNP Identification and Genotyping

The reference genome data for *A. cornea* were obtained from the NCBI database (https://www.ncbi.nlm.nih.gov/bioproject/PRJNA943604/, accessed on 31 July 2023). High-quality sequencing reads (clean reads), generated after trimming with Trimmomatic (v0.36), were aligned to the reference genome using BWA (v.0.7.17), producing sequence alignment map (SAM) files. The SAM files were subsequently sorted and merged using SAMtools (v.1.10), and duplicate reads were removed using Picard (v1.92) [23]. Based on the alignment results, sequencing depth and genome coverage were calculated using a custom Perl script. The resulting BAM files were subjected to SNP detection using the HaplotypeCaller module of GATK (v4.1.2.0) [24], generating Variant Call Format (VCF) files. Variant quality filtering was performed using BCFtools and VCFtools with the following parameters: QD < 2.0||FS > 60.0||MQ < 40.0||SOR > 10.0. Detected variants were functionally annotated using ANNOVAR (version released on 8 June 2020). High-quality SNPs were selected from the entire dataset based on the following criteria: average coverage depth > 5×, minor allele frequency (MAF) > 0.05, missing rate < 0.2, heterozygosity < 0.5, and average quality value (AverageQ) > 20. After filtering, a total of 2,512,778 high-quality SNPs were retained for subsequent analyses. Using gcta (version 1.93.2), principal component analysis (PCA) was performed to assess the genetic structure of the *A. cornea* germplasm resources [25]. The raw sequencing reads have been submitted to the NCBI Sequence Read Archive (SRA) under accession number PRJNA1406526.

### 2.5. Genome-Wide Association Study (GWAS)

GWAS is a powerful approach for identifying associations between genotypes and phenotypes across the entire genome and is widely used to uncover candidate genes underlying phenotypic traits [26]. Based on phenotypic datasets of 10 fruiting body-related traits (FBRTs) surveyed in 2024 and 2025 and whole-genome SNP data from 140 strains, GWAS was performed using the mixed linear model (LMM) implemented in GEMMA software (v0.98.1) [27]. The kinship matrix (K), calculated using the centered relatedness method, was included as a random effect to control for population structure and relatedness among individuals. The genome-wide significance threshold for FBRTs was determined by calculating the expected threshold for significant SNPs based on the total number of SNP markers [28]. The expected threshold was set as −log_10_(P) > −log_10_(0.05/2,512,778) ≈ 7.7, where P = 0.05/n and n represents the number of SNPs (2,512,778 in this study), corresponding to the Bonferroni correction for multiple testing. For certain traits, no significant SNPs were identified under this stringent threshold. Therefore, to obtain potentially informative loci, a suggestive threshold of −log_10_(P) ≥ 5.5 was additionally applied. If a significant FBRT-associated SNP was located within a gene sequence or its flanking noncoding region, the corresponding gene was considered a candidate FBRT-associated gene. FBRT-associated genes identified by GWAS and candidate genes related to genetic differentiation detected through genome-wide scans were collectively defined as candidate genes associated with phenotypic differentiation.

### 2.6. Candidate Gene Annotation

SNP markers significantly associated with target traits were mapped onto the reference genome, and their corresponding confidence intervals were determined to screen potential trait-related candidate genes. Subsequently, these candidate genes were functionally annotated by comparison with multiple databases, including the Non-Redundant Protein Database (NR), the Kyoto Encyclopedia of Genes and Genomes (KEGG), and Gene Ontology (GO).

### 2.7. Haplotype Analysis of Significant GWAS Loci

Based on the significant loci identified by GWAS, haplotype analysis was performed for these significantly associated SNPs using Haploview software (v.4.2) [29]. According to different allelic variants, the 140 *A. cornea* strains were classified into distinct groups, and bar plots of phenotypic traits corresponding to different haplotype types were generated using R software (v.4.3.1).

## 3. Results

### 3.1. Phenotypic Diversity of Quantitative Traits in A. cornea

**Figure 1:**
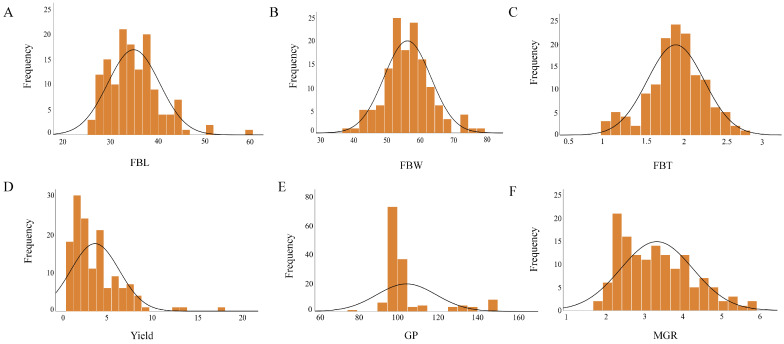
Statistics of quantitative traits of *A. cornea*. (**A**) Fruiting body length (FBL);(**B**) Fruiting body width (FBW); (**C**) Fruiting body thickness (FBT); (**D**) Yield; (**E**) Growth period(GP); (**F**) Mycelial growth rate (MGR).

The distribution patterns of six quantitative traits in *A. cornea* were statistically analyzed, and normal distribution curves were fitted for each trait. The results indicated that the experimental population exhibited a high level of phenotypic variation, providing a rich basis of genetic variation for subsequent association analyses (Figure 1). In addition, the minimum, maximum, mean, standard deviation (SD), and coefficient of variation (CV) of these traits were systematically summarized and analyzed (Table 1). A comparative analysis of CV values showed that yield exhibited the highest coefficient of variation, with values of 0.94 in 2024 and 0.83 in 2025. This was followed by MGR, with a CV of 0.32, which was evaluated only in 2024. The CV of FBL remained consistent between the two years, whereas yield showed the greatest interannual difference in CV. Among the six quantitative traits, the mean CV values across the two years followed the order yield > MGR > FBT > FBL > GP > FBW, indicating that yield and ear thickness displayed relatively abundant phenotypic variation within the population.

### 3.2. Diversity Analysis of Qualitative Traits in A. cornea

As shown in Table 2, the Shannon–Wiener diversity index (H′) of four qualitative traits in *A. cornea* ranged from 0.786 to 1.150. Among these traits, DCF exhibited the highest H′ value (1.150). Frequency distribution analysis showed that four phenotypic categories of DCF, namely white, grayish white, grayish yellow, and brownish yellow, accounted for 9.29%, 8.21%, 46.43%, and 35.71%, respectively. The relatively dispersed distribution indicates that this trait exhibits a high level of genetic variation. Meanwhile, the Simpson diversity index (D) of DCF was also relatively high (0.642), indicating that this trait comprises multiple categories with a relatively even distribution and serves as an important qualitative trait for distinguishing phenotypic differences among accessions.

### 3.3. Correlation Analysis of Agronomic Traits in A. cornea

**Figure 2:**
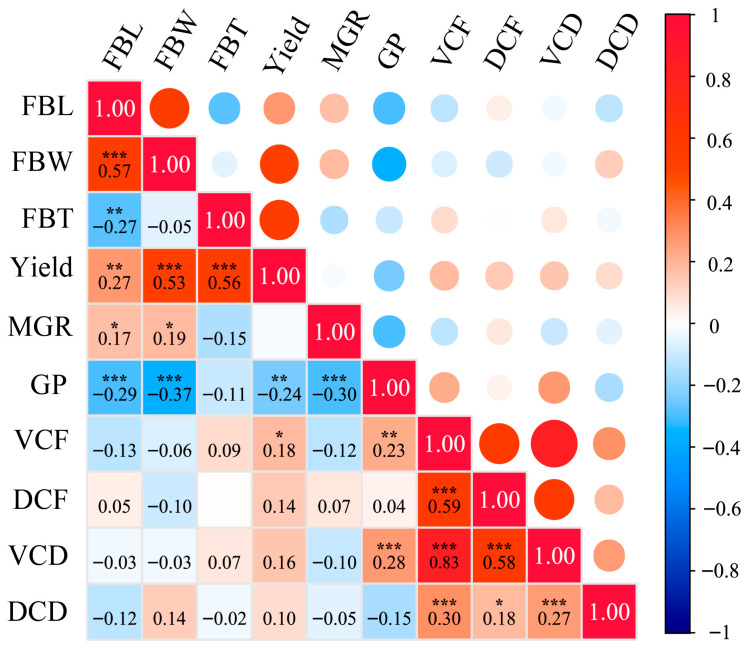
Correlation analysis of ten phenotypic traits across 140 *A. cornea* germplasm accessions. * *p* ≤ 0.05 ** *p* ≤ 0.01 *** *p* ≤ 0.01.

Correlation analysis of ten agronomic traits across 140 *A. cornea* accessions revealed that significant correlations were prevalent among these traits (Figure 2). MGR was significantly positively correlated with FBL and FBW, whereas it was significantly negatively correlated with GP. During fruiting body development, FBL showed significant positive correlations with FBW, yield, and MGR, but significant negative correlations with FBT and GP. Yield was significantly positively correlated with FBL, FBW, FBT, and VCF, but significantly negatively correlated with GP. Significant positive correlations were also observed among color-related traits, including VCF, DCF, VCD, and DCD.

### 3.4. Genome-Wide Association Analysis

**Figure 3:**
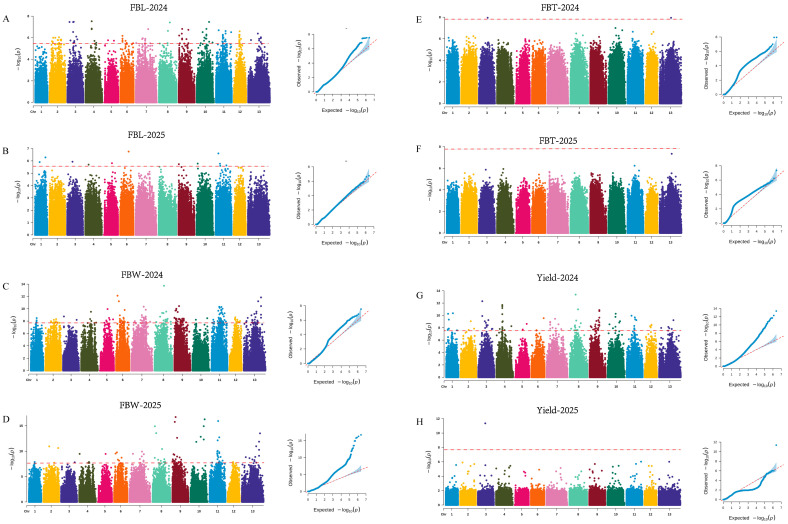
Manhattan and Q–Q plots for yield-related traits in *A. cornea*. (**A**,**B**) Fruiting body length; (**C**,**D**) fruiting body width; (**E**,**F**) fruiting body thickness; (**G**,**H**) yield.

**Figure 4:**
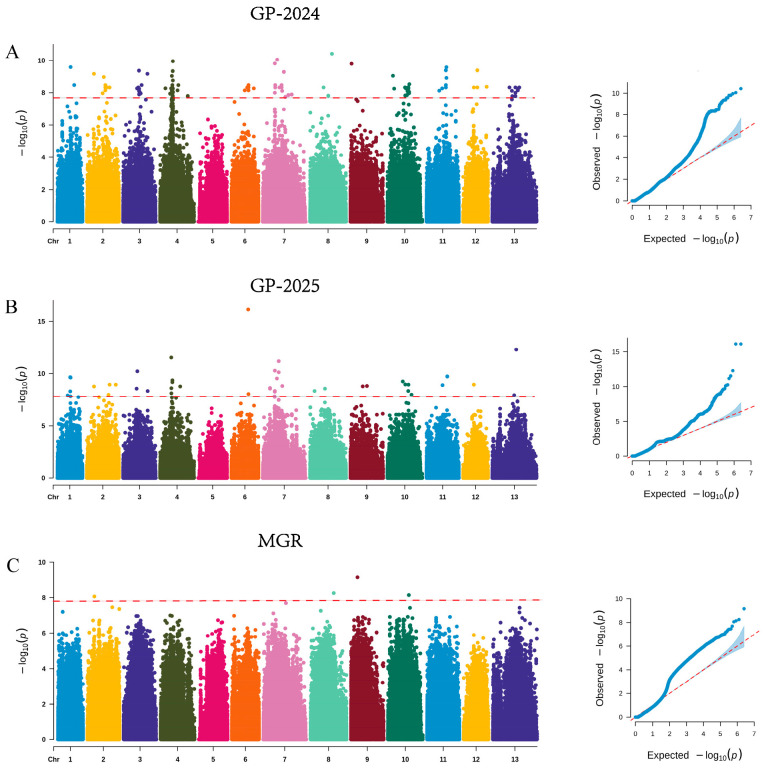
Manhattan and Q–Q plots for MGR and GP traits in *A. cornea*. (**A**,**B**) Growth period; (**C**) mycelial growth rate.

In the two-year GWAS of FBL, 104 and 14 suggestive SNPs were identified, respectively, and the SNP consistently detected across both years was located on chromosome 9. For FBW, 214 and 99 significant SNPs were identified in the two respective years, among which 19 SNPs were consistently detected, predominantly distributed on chromosome 9. For FBT, 2 and 0 significant SNPs were identified in the two years, and these loci were located on chromosomes 3 and 13. For yield, 101 and 1 significant SNPs were identified in the two years, mainly distributed across chromosomes 9, 4, and 3 (Figure 3; Table S4). A total of four significant SNPs associated with MGR were identified, located on chromosomes 2, 8, 9, and 10. For GP, 114 and 45 significant SNPs were identified in the two respective years, among which one SNP (chr1-2459000) was consistently detected across both years (Figure 4; Table S4).

**Figure 5:**
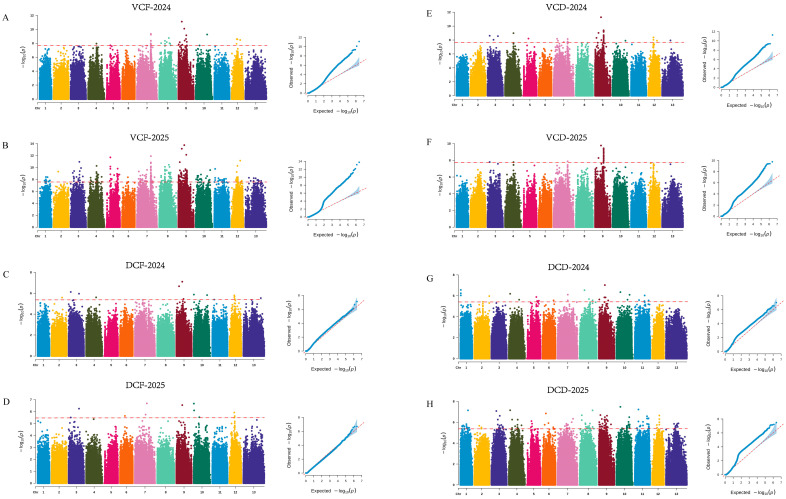
Manhattan and Q–Q plots for fruit-body color related traits in *A. cornea*. (**A**,**B**) Predominant ventral color of fresh ear pieces; (**C**,**D**) dorsal color of fresh ear pieces; (**E**,**F**) ventral color of dried ear pieces; (**G**,**H**) dorsal color of dried ear pieces.

In the association analyses of color-related traits, 34 and 353 SNPs were significantly associated with VCF, 38 and 25 SNPs with VCD, 12 and 11 SNPs were suggestively associated with DCF, and 27 and 134 SNPs were suggestively associated with DCD in the two years (Figure 5; Table S4). Further comparison between the two years revealed that 34 SNPs were consistently associated with VCF, 6 SNPs with DCF, 24 SNPs with VCD, and 16 SNPs with DCD. Among the consistently detected loci, chr7-5469460 and chr9-1365495 were shared between VCF and DCF, chr3-809516 was shared between DCF and VCD, and chr9-2327432 was repeatedly detected in VCF, DCF, and VCD.

### 3.5. Gene Annotation

Based on the GWAS results, candidate genes were identified within ±5 kb flanking regions of significant SNPs located in genic regions. A total of 42 putative candidate genes were identified across ten important agronomic traits in *A. cornea* (Table S5). Functional annotation analyses revealed that candidate genes associated with different traits exhibited distinct functional profiles. For MGR, candidate genes mainly encoded proteins involved in post-translational modification and metabolic processes, such as the glycoside hydrolase, complex subunit STT3 and acidic proteases. These genes were primarily associated with protein glycosylation, protein hydrolysis, nucleotide hydrolysis, and carbohydrate metabolism. For GP, candidate genes mainly encoded Osmotin/thaumatin-like proteins and glucan 1,3-β-glucosidase, which are involved in transcriptional regulation, signal transduction, and cell cycle-related processes. For yield related traits, candidate genes were mainly annotated as glycoside hydrolase family proteins (GH20) and were involved in cell wall polysaccharide metabolism, energy metabolism and redox regulation, as well as intracellular transport. Candidate genes associated with fruiting body morphological traits (e.g., FBL, FBW, and FBT) were mainly annotated as 1,3-β-glucanases, glycoside hydrolases, and Lys1p, which are closely related to cell wall polysaccharide degradation and redox regulation. In addition, candidate genes associated with fruiting body color traits, such as cytochrome P450, were mainly involved in pigment biosynthesis and modification, aromatic compound metabolism, redox reactions, and material transport.

### 3.6. Haplotype Analysis

**Figure 6:**
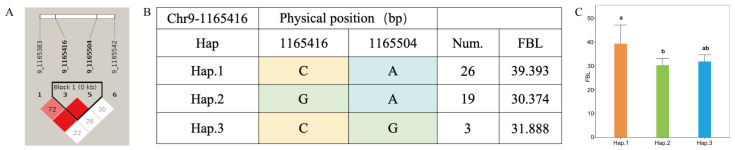
Haplotype analysis of Chr9-1165416 and phenotypes of FBL among different haplotypes in *A. cornea*. (**A**) Linkage region associated with FBL; (**B**) haplotypes identified within the linkage region; (**C**) bar plot showing (Different letters a, b indicate significant differences among groups at *p* < 0.05 based on multiple comparison test. FBL among accessions carrying the three haplotypes.

Haplotype analysis was performed for significant loci associated with quantitative traits in *A. cornea*, and analysis of variance of phenotypic data revealed that the numbers of accessions carrying different haplotypes varied among traits, with superior haplotypes exhibiting significantly enhanced phenotypic performance in their corresponding traits. The FBL associated SNP Chr9-1165416, together with one adjacent SNP, defined a linkage region (Figure 6A). Three haplotypes were identified within this region, among which 26 accessions carried haplotype-CA (Hap.1) (Figure 6B). Accessions harboring Hap.1 exhibited a significantly greater mean fruiting body length than those carrying the other two haplotypes, indicating that haplotype-CA represents a superior haplotype for FBL (Figure 6C).

**Figure 7:**
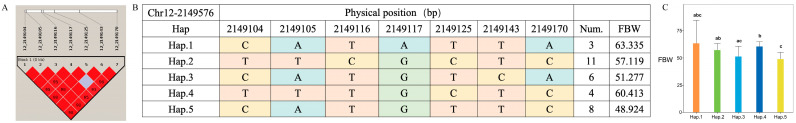
Haplotype analysis of Chr12-2149576 and phenotypes of FBW among different haplotypes in *A. cornea*. (**A**) Linkage region associated with FBW; (**B**) haplotypes identified within the linkage region; (**C**) bar plot (Different letters a, b, c indicate significant differences among groups at *p* < 0.05 based on multiple comparison test.) showing FBW among accessions carrying the five haplotypes.

The FBW-associated SNP Chr12-2149576, together with seven surrounding SNPs, defined a linkage region (Figure 7A). Five haplotypes were identified within this region, among which three accessions carried haplotype-CATATTA (Hap.1) (Figure 7B). Accessions harboring Hap.1 exhibited a significantly greater mean fruiting body width than those carrying the other four haplotypes, indicating that haplotype-CATATTA represents a superior haplotype for FBW (Figure 7C).

**Figure 8:**
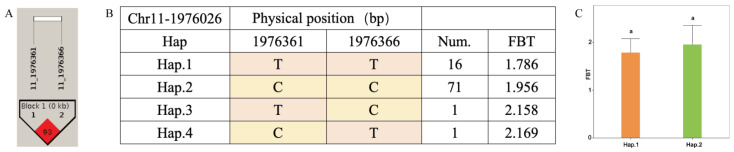
Haplotype analysis of Chr11-1976026 and phenotypes of FBT among different haplotypes in *A. cornea*. (**A**) Linkage region associated with FBT; (**B**) haplotypes identified within the linkage region; (**C**) bar plot showing FBT among accessions carrying the two haplotypes. The same letter a indicates no significant difference among groups (*p* > 0.05).

The FBT-associated SNP Chr11-1976026, together with two adjacent SNPs, defined a linkage region (Figure 8A). Four haplotypes were identified within this region: haplotype-TT (Hap.1), haplotype-CC (Hap.2), haplotype-TC (Hap.3), and haplotype-CT (Hap.4). Among the 140 accessions of *A. cornea*, 16 carried Hap.1 and 71 carried Hap.2, whereas only one accession carried Hap.3 or Hap.4, respectively (Figure 8B). Due to the limited number of accessions carrying Hap.3 and Hap.4, which precluded robust statistical analysis, only Hap.1 and Hap.2 carrying accessions were included in the analysis of variance. The results showed that accessions carrying Hap.1 exhibited a higher mean FBT than those carrying Hap.2, although the difference was not statistically significant (Figure 8C).

**Figure 9:**
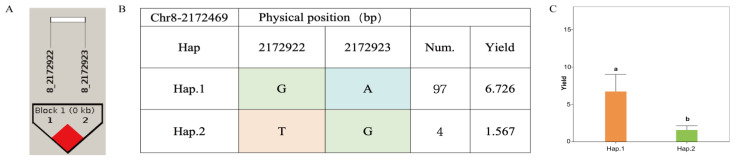
Haplotype analysis of Chr8-2172469 and phenotypes of yield among different haplotypes in *A. cornea*. (**A**) Linkage region associated with yield; (**B**) haplotypes identified within the linkage region; (**C**) bar plot (Different letters (a, b) indicate significant differences among groups at *p* < 0.05 based on multiple comparison test.) showing yield among accessions carrying the two haplotypes.

The yield associated SNP Chr8-172469, together with two adjacent SNPs, defined a linkage region (Figure 9A). Two haplotypes were identified within this region: haplotype-GA (Hap.1) and haplotype-TG (Hap.2). Among the 140 accessions of *A. cornea*, 97 carried Hap.1, whereas only four carried Hap.2 (Figure 9B). Accessions harboring Hap.1 exhibited a significantly higher mean yield than those carrying Hap.2, indicating that haplotype-GA represents a superior haplotype for yield (Figure 9C).

The GP-associated SNP Chr1-2459000, together with three adjacent SNPs, defined a linkage region (Figure S1A). Two haplotypes were identified within this region: haplotype-CTC (Hap.1) and haplotype-TGT (Hap.2) (Figure S1B). Among the 140 accessions of *A. cornea*, 83 accessions carried Hap.1, and accessions harboring Hap.1 exhibited a significantly superior phenotypic performance for growth period compared with those carrying Hap.2. These results indicate that haplotype-CTC (Hap.1) represents a superior haplotype associated with GP (Figure S1C). For the MGR associated SNP Chr9-1207827, together with two adjacent SNPs, defined a linkage region (Figure S2A). Three haplotypes were identified within this region: haplotype-CG (Hap.1), haplotype-CA (Hap.2), and haplotype-TG (Hap.3) (Figure S2B). Among the 140 accessions, 15 carried Hap.1, and accessions harboring Hap.1 exhibited a significantly higher mycelial growth rate than those carrying Hap.2 or Hap.3. These results indicate that haplotype-CG (Hap.1) represents a superior haplotype for mycelial growth rate (Figure S2C).

## 4. Discussion

### 4.1. Phenotypic Evaluation of A. cornea Germplasm

*A. cornea* exhibits a relatively low level of morphological differentiation and a limited number of phenotypic traits. Effective germplasm evaluation therefore relies on the use of materials with broad genetic backgrounds and wide ranges of phenotypic variation, which provide a reliable basis for cultivar breeding and genetic improvement [23]. Phenotypic diversity analysis of germplasm resources is an effective approach for identifying and screening elite or unique germplasm [24]. Phenotypic diversity assessments have been widely applied in germplasm evaluation of crops and edible fungi, including maize, sorghum, sugarcane, *Morchella esculenta*, and *Auricularia heimuer* [30,31,32,33,34]. Phenotypic diversity is commonly quantified using indices such as the coefficient of variation (CV), the Shannon–Wiener index (H′), and the Simpson index (D), each reflecting distinct aspects of germplasm diversity. Among these indices, CV reflects the extent of phenotypic variation within a population and provides an intuitive measure of trait differentiation and evolutionary or breeding potential [35]. In the present study, the CV values of fruiting body size traits (FBL, FBW, and FBT) ranged from 0.14 to 0.27, which are comparable to those reported by Cao for *A. cornea* (0.31–0.40) [20] and for the congeneric species *Auricularia heimuer* (0.19–0.22) [36]. H′ reflects the richness and evenness of different phenotypic traits [37], whereas the Simpson index (D) is primarily used to evaluate the dominance of major phenotypic categories within a population [38]. For qualitative traits, the H′ values of the four traits ranged from 0.786 to 1.150, indicating substantial phenotypic polymorphism in appearance-related traits within the population. Among these traits, the DCF exhibited the highest H′ value (1.15) and a relatively high Simpson index (D = 0.642). This trait comprised multiple color categories, including white, grayish white, grayish yellow, and brownish yellow, with relatively even frequency distributions, suggesting a high level of genetic variation and highlighting its importance as a key phenotypic trait for discriminating among accessions.

Correlation analysis of the ten agronomic traits across the 140 accessions revealed that these traits are not independent but instead exhibit varying degrees of coordinated variation and trade-offs. During fruiting body development, FBL showed significant positive correlations with FBW, yield, and MGR while being negatively correlated with FBT and the GP. These relationships suggest that fruiting body morphogenesis does not involve the synchronous enhancement of all morphological dimensions but rather reflects a coordinated allocation of morphological resources. Specifically, an increase in lateral expansion (FBL and FBW) is often accompanied by a reduction in vertical thickening, a pattern consistent with previous findings reported by Anran Xu [39]. Such observations indicate the presence of coordination–constraint relationships among different morphological dimensions, which together shape the fruiting body phenotype and indirectly influence yield formation. In addition, strong positive correlations were detected among color-related traits, including VCF, DCF, VCD, and DCD. This pattern may be attributable to pleiotropic effects of individual genes or tight linkage among loci controlling these traits, resulting in their coordinated expression in *A. cornea* [40].

### 4.2. Genome-Wide Association Analysis of Agronomic Traits in A. cornea

Principal component analysis (PCA) was performed based on genome-wide SNP markers to evaluate the genetic structure of the association panel. The first two principal components explained 31.55% and 21.18% of the total genetic variation, respectively (Figure S3). In the genome-wide association analyses of yield-related quantitative traits, the number of significant loci detected and the degree of overlap between years varied markedly among traits, indicating that their underlying genetic architectures and sensitivities to environmental factors are not uniform. Notably, no overlapping significant loci were detected across the two years for FBT and yield. This result suggests that the genetic signals underlying these traits may exhibit stronger environmental dependence or more complex genetic architectures, with phenotypic variation likely governed by multiple loci with small effects. Such traits are therefore more susceptible to annual climatic variation, cultivation conditions, and micro-environmental factors during development, which can substantially reduce the reproducibility of significant association signals across years. Similar observations have also been reported in *Lentinula edodes*, where yield-related traits displayed limited interannual consistency in GWAS results [41]. Alternatively, the absence of repeatedly detected loci may be partially attributable to the limited population size evaluated in each year, which may not have been sufficient to capture the full spectrum of genetic variation present in *A. cornea* germplasm.

The repeatedly detected loci for FBL and FBW across the two years, as well as a substantial proportion of significant loci associated with yield, were predominantly enriched on chromosome 9, suggesting that chr9 may have key genomic regions involved in fruiting body morphogenesis and yield formation. Notably, significant loci associated with MGR were also distributed on chr9. When considered together with the correlation analysis revealing strong positive associations among yield, FBL, FBW, and MGR, the enrichment of loci on chr9 is likely indicative of pleiotropic regulation or tightly linked quantitative trait loci (QTL) clusters. These genomic regions may therefore coordinately regulate fruiting body morphological development and yield formation in *A. cornea*.

Based on functional annotation of candidate genes underlying significant loci associated with agronomic traits in *A. cornea*, this study revealed clear functional differentiation among genes associated with different traits. Candidate genes related to mycelial growth rate were primarily involved in protein processing and degradation, energy metabolism, and intracellular transport. Among these, genes encoding subtilisin-like proteases and α/β hydrolases may participate in extracellular or intracellular protein processing and turnover [42,43], thereby providing the metabolic and structural basis required for rapid mycelial extension.

Candidate genes associated with fruiting body morphological traits, including FBL, FBW, and FBT, were predominantly annotated to functions related to cell wall polysaccharide metabolism, maintenance of cellular structure, and material transport. For example, exo-β-1,3-glucanase is involved in the degradation and remodeling of cell wall glucans [44], which may influence cell expansion capacity and tissue compactness during fruiting body development. As yield represents a typical complex quantitative trait, its associated candidate genes exhibited prominent enrichment in primary metabolism and energy-related processes. Functional annotations of genes encoding pyruvate carboxylase, PLP-dependent transferases, and multiple members of the α/β hydrolase family suggest that the efficiency of carbon and nitrogen metabolism may indirectly determine fruiting body biomass accumulation by regulating nutrient assimilation and conversion. Collectively, these findings indicate that the formation of fruiting body morphological traits is governed not only by cell growth rate but also by dynamic cell wall remodeling and intracellular transport efficiency, which is consistent with previous GWAS results reported in *Lentinula edodes* [45]. Candidate genes associated with the GP were mainly involved in signal regulation and cellular process control, such as those encoding proteins containing RhoGAP domains. RhoGAP proteins are known to regulate small GTPase signaling pathways in eukaryotes and are closely associated with cell morphology changes and developmental phase transitions [46]. These annotations suggest that variation in growth period among *A. cornea* accessions may arise from fine-scale regulation of developmental progression by signal transduction networks. In addition, candidate genes associated with color-related traits, including cytochrome P450s, atypical manganese peroxidases (MnPs), and NAD(P)-binding proteins, were functionally characterized by enrichment in redox reactions, secondary metabolism, and cellular structural regulation. Oxidative modification and electron transfer processes may therefore play critical roles in pigment biosynthesis and transformation. Among these, cytochrome P450 enzymes have been widely reported to be involved in pigment deposition in both plants and edible fungi [47,48]. Some of the candidate genes represent typical fungal functions and have been identified as candidate genes in basidiomycetes and other fungi (Table S6). Therefore, these genes should be considered as primary candidate genes. Given the widespread distribution of these gene families in fungi, their functional roles in the traits under study still need to be validated.

### 4.3. Analysis of Superior Haplotypes Associated with Agronomic Traits in A. cornea

A haplotype refers to a group of closely linked SNPs located on the same chromosome or within a specific genomic region. Haplotype analysis facilitates a deeper understanding of allelic distribution patterns and the genetic relationships between genotypes and phenotypes [49]. As an important validation and refinement step following GWAS, haplotype analysis enables the interpretation of association signals to be elevated from individual significant SNPs to combinations of allelic variants within linkage blocks, thereby providing a representation that more closely reflects the true genetic architecture of complex traits. Previous studies have demonstrated that haplotype-based comparisons within linkage disequilibrium (LD) blocks can substantially enhance the explanatory power for phenotypic variation and provide more stable genetic units for marker-assisted selection. This strategy has been widely adopted as an important complement to GWAS in crops such as wheat [50,51]. The pattern in which superior haplotypes exhibit significantly enhanced phenotypic performance compared with alternative haplotypes is commonly observed in studies of disease resistance, yield, and morphological traits in crops. Such patterns are generally interpreted as evidence that functional variants, or markers tightly linked to functional variants, reside within the corresponding linkage blocks. Through long-term natural or artificial selection, favorable allelic combinations become enriched, ultimately forming “superior haplotypes” that can be directly exploited in breeding programs. For example, the introgression of a superior haplotype harboring high expression of PibH8 into rice cultivars has been shown to significantly enhance drought tolerance and resistance to rice blast disease [52]. In cultivated populations of *A. cornea*, long-term selection may have preferentially favored phenotypes characterized by larger and wider fruiting bodies, facilitating the accumulation of superior haplotypes associated with these traits. In the present study, linkage blocks associated with FBL, FBW, and yield consistently harbored haplotypes that exhibited both significantly superior phenotypic performance and relatively high frequencies within the population. These observations suggest that these genomic regions may have undergone prolonged artificial selection, leading to the enrichment of favorable allelic combinations. This pattern is consistent with the reshaping of allele frequency distributions at loci controlling key commercial traits during the domestication and improvement of edible fungi [53].

## 5. Conclusions

The genetic basis of important agronomic traits in *A. cornea* was systematically investigated using a diverse panel of 140 accessions evaluated over two consecutive years. Significant genetic variation was observed for all traits, and relatively high heritability was detected for most of them. Genome-wide association analysis revealed multiple SNPs significantly associated with key agronomic traits. Notably, yield-related loci were enriched on chromosome 9, suggesting that this region may play an important role in yield formation. Through the integration of statistical significance thresholds and linkage disequilibrium information, ten core associated SNPs and forty-two putative candidate genes were identified. These genes were predominantly involved in cell wall metabolism, oxidative regulation, pigment biosynthesis, and signal transduction pathways. Haplotype analysis further identified five favorable haplotypes. Accessions carrying these haplotypes exhibited significant phenotypic advantages in the corresponding traits, indicating their potential utility for molecular breeding applications. Despite these findings, several limitations must be acknowledged. Due to the current lack of a mature genetic transformation system in *A. cornea*, direct functional validation of candidate genes could not be performed in this study. The future establishment of an efficient and stable transformation platform, combined with multi-omics integration analyses, will facilitate a deeper understanding of the regulatory relationships among candidate genes. In addition, an expansion of the population size and the incorporation of more genetically diverse germplasm resources would enhance mapping resolution and statistical power. Furthermore, multi-year and multi-environment trials are necessary to validate the genetic stability and practical breeding value of the identified favorable haplotypes. Future studies that integrate functional genomics with marker-assisted selection strategies are expected to facilitate the precise genetic improvement of *A. cornea*.

## Figures and Tables

**Table 1:** Statistical analysis of six quantitative characteristics of *A.* *cornea*.

Trait	Year	Min	Max	Mean	SD	CV
MGR	2024	1.38	5.98	3.17	1.01	0.32
GP	2024	73.00	145	99.99	14.77	0.15
	2025	64.00	118.00	75.96	12.98	0.17
Yield	2024	1.23	25.42	7.13	6.72	0.94
	2025	0.11	16.80	3.21	2.66	0.83
FBL	2024	24.23	60.06	35.39	6.13	0.17
	2025	20.39	56.98	34.03	5.91	0.17
FBW	2024	37.36	100.78	55.20	7.98	0.14
	2025	37.36	93.37	54.10	7.96	0.15
FBT	2024	0.90	4.83	1.76	0.47	0.27
	2025	0.97	2.60	1.87	0.38	0.20

**Table 2:** Frequency distribution of four characteristics.

Trait	Frequency (%)						
	1	2	3	4	5	H′	D
VCF	7.14	0.71	12.14	12.86	67.14	1.011	0.513
DCF	9.29	8.21	46.43	35.71		1.150	0.642
VCD	7.14	2.50	2.50	19.29	68.57	0.949	0.486
DCD	8.57	70.36	21.07			0.786	0.453
FBT	7.14	0.71	12.14	12.86	67.14	1.011	0.513

## Data Availability

The raw sequencing data have been deposited in the NCBI Sequence Read Archive (SRA) under the accession number PRJNA1406526.

## Supplementary Materials

The following supporting information can be downloaded at https://www.mdpi.com/article/10.3390/jof12030186/s1, Table S1: Information of 140 *A. cornea* stains. Table S2: Abbreviations. Table S3: Agronomic characters index of *A. cornea* strains and evaluation methods. Table S4: SNP loci significantly associated with agronomic traits. Table S5: Functional annotation of key candidate genes associated with traits. Table S6: Major candidate (supported by literature) genes associated with agronomic traits in *A. cornea*. Figure S1: Haplotype analysis of Chr1-1976026 and phenotypes of GP among different haplotypes. (A) Linkage regions associated with GP. (B) Haplotype; (C) GP bar chart of two haplotype materials. Figure S2: Haplotype analysis of Chr9-1207827 and phenotypes of MGR among different haplotypes. (A) Linkage regions associated with MGR. (B) Haplotype. (C) MGR bar chart of three haplotype materials. Figure S3: Principal component analysis (PCA).
